# JK5G postbiotics attenuate immune-related adverse events in NSCLC patients by regulating gut microbiota: a randomized controlled trial in China

**DOI:** 10.3389/fonc.2023.1155592

**Published:** 2023-08-04

**Authors:** Mengting Chen, Liling Ma, Huiqing Yu, Shaoyi Huang, Junhui Zhang, Juan Gong, Liejun Yang, Lan Chen, Haojun Luo, Ling Tian, Sixiong Wang

**Affiliations:** ^1^ Department of Clinical Nutrition, Chongqing University Cancer Hospital, School of Medicine, Chongqing University, Chongqing, China; ^2^ Department of Geriatric Oncology and Department of Palliative Care, Chongqing University Cancer Hospital, School of Medicine, Chongqing University, Chongqing, China

**Keywords:** postbiotics, gut microbiota, immune-related adverse events, non-small-cell lung cancer, enterotype

## Abstract

**Scope:**

This study aimed to evaluate the effects of JK5G postbiotics to regulate imbalanced gut microbiota and its impacts on the efficacy and incidence rate of immune-related adverse events (irAEs) in non-small-cell lung cancer (NSCLC) patients treated with immune checkpoint inhibitors (ICIs).

**Methods:**

This randomized, double-blind, placebo-controlled trial was conducted in China and included non-squamous or squamous NSCLC patients without EGFR, ROS1, and ALK alteration, treatment-naive, and stage IIIb-IV. Patients were randomly (1:1) divided into two groups to receive four cycles (three weeks for each cycle) of programmed cell death-1 (PD-1) plus chemotherapy plus placebo (control group, n = 30) or to receive PD-1 plus chemotherapy plus JK5G postbiotics (JK5G group, n = 30). The primary endpoint was objective response rate. The secondary endpoints were quality of life (QoL), adverse effects, and the 16S DNA sequencing of gut microbiota, blood inflammatory cytokines, and lymphocyte subsets. This study was registered at www.chictr.org.cn (ChiCTR2200064690).

**Results:**

Sixty patients were enrolled. The objective response rate was 36.67% (11/30) in the control group and 50.00% (15/30) in the JK5G group (*p* = 0.297). The JK5G group had better QoL and nutritional levels, as well as lower depression symptoms than the control group (all *p* < 0.05). Moreover, the JK5G group had a lower incidence of anemia (63.33% vs. 13.33%, *p* < 0.001), decreased lymphocyte count (20.00% vs. 0%, *p* = 0.010), decreased appetite (53.33% vs. 16.67%, *p* = 0.003), nausea (33.33% vs. 6.67%, *p* = 0.010), and asthenia (30.00% vs. 6.67%, *p* = 0.017) than the control group. Moreover, JK5G attenuated gut microbiota imbalance, accompanied by increased *Faecalibacterium*, *Ruminococcaceae*, and fecal butyrate concentration, and diminished *Escherichia-Shigella*. Furthermore, JK5G administration significantly decreased the levels of pro-inflammatory markers, including TNF-α, IL-2, and C-reactive protein (CRP) (all *p* < 0.05). Significant increases in CD3^+^CD4^+^ T cells and CD4/CD8 ratio were observed in the peripheral blood of JK5G group patients (all *p* < 0.05). The enterotype data showed that patients were clustered into *Blautia* (E1) and *Escherichia-Shigella* (E2) enterotypes, and JK5G postbiotics intervention might be related to enterotype modulations.

**Conclusion:**

Our current findings indicated that JK5G postbiotics might attenuate irAEs, and enhance the QoL and nutrition levels of advanced NSCLC patients who received ICIs. JK5G postbiotics could also improve the gut microbiota structures and ameliorate the tumor microenvironment and inflammation.

**Clinical trial registration:**

www.chictr.org.cn, identifier ChiCTR2200064690.

## 1 Introduction

A paradigm shift has occurred in the management of non-small-cell lung cancer (NSCLC) due to advances in cancer immunotherapy, including immune checkpoint inhibitors (ICIs) that target programmed cell death-1 (PD-1) and programmed cell death-ligand 1 (PD-L1) (1, 2). Cancer patients worldwide receive these strategies as standard treatment options, but only a minority respond to ICIs clinically (3, 4). The clinical prospect of ICIs is hindered by severe immune-related adverse events (irAEs) (5, 6). Therefore, identifying strategies to improve the efficacy of cancer immunotherapy and decrease irAEs is essential. Emerging evidence has shown that the intestinal microbiome can play a fundamental role in response to ICIs (7, 8). For example, a recent study found that *Ruminococcaceae* and *Faecalibacterium* significantly increased in ICIs-responding patients and enhanced the host anti-tumor immune response (3). Furthermore, researchers have recently reported a difference in the intestinal microbiome between advanced NSCLC patients receiving ICIs treatment with low- and high-level irAEs (9). Hence, modulating the gut microbiome with products and management approaches, including postbiotics, probiotics, prebiotics, and dietary intervention, might be a potential strategy to modulate immunotherapy responses (10, 11). According to the definition, postbiotics are inactivated commensal bacteria with beneficial effects on the host and are composed of inanimate microorganisms (12, 13). Probiotics and postbiotics might help reduce the occurrence of cancer therapy-related side effects (11). The gut microbiota modulates the response to cancer therapy and susceptibility to toxic side effects. Multiple mechanisms, including regulation of the cytotoxic activity, humoral responses, and inflammatory reaction, have been proposed to be involved in irAEs (9). The available reports indicate possible mechanisms behind the protection of probiotics and postbiotics on immunotherapy toxicity, such as inhibiting proinflammatory cytokines (9, 11). Probiotics or postbiotics can improve the gut microbial population, increase mucus secretion, and prevent the destruction of tight junction proteins (11, 13). Furthermore, decreased gut dysbiosis and intestinal leakage after probiotic therapy might minimize the development of inflammatory biomarkers and blunt unnecessary immune system activation (9, 11). In turn, probiotics improve the differentiation of T-cells against Th2 and the development of Th2 cytokines such as IL-4 and IL-10 (9, 11). However, probiotics are not completely safe. They can trigger systemic infections, damaging metabolic activities, and excessive immunological stimulation in susceptible people (14, 15). Moreover, postbiotics have several benefits compared to live probiotics (14, 15). First, postbiotics are safer for weak individuals, including immunocompromised, old, and hospitalized, without the risk of possible infections (15). Other significant advantages of postbiotics are longer shelf-life and stable storage features compatible with less developed areas, distinct chemical structures, more safety dose factors, promising adsorption and secretion ability, suitable to reach distant organs of the body (15). Generally, postbiotics possess the health advantages of probiotics while minimizing the associated risk factors of using live microorganisms (15, 16). A study reported that JK5G postbiotics could slow the development of colorectal cancer and might regulate the tumor microenvironment via gut microbiota changes and metabolite bands on different pathways (17). Postbiotics are a potential novel therapeutic strategy to modulate immune therapy (11). However, the effects of postbiotics on NSCLC immunotherapy remain unknown. Therefore, we choose JK5G postbiotics to treat NSCLC patients and answer these questions.

The human gut microbiota can be divided into three un-nation or continent-specific enterotypes (18). Although the stratification strategy of gut enterotypes is still controversial (19), several lines of evidence have shown that cancer patients in different enterotypes might have different gut microbiota responses under the same treatment (19–22). Hence, more longitudinal studies are needed to clarify gut microbial complexity and its patterns in cancer control and identify its biological and clinical significance. To approach this question, we focused on whether the intervention effects of JK5G postbiotics were correlated with specific gut enterotypes. These findings supported that administering JK5G postbiotics might be a prospective strategy to decrease the irAEs of ICIs and ameliorate the treatment tolerance via gut microbiota regulation.

Therefore, in the present study, we evaluated the effects of JK5G postbiotics on regulating the imbalanced gut microbiota and its impact on tumor efficacy and incidence rate of irAEs in NSCLC patients treated with ICIs.

## 2 Methods

### 2.1 Patient enrollment

From June 10, 2021, to August 1, 2022, we conducted a randomized, double-blind, placebo-controlled trial at Chongqing University Cancer Hospital in Chongqing, China. We included individuals aged 40-70 with histologically or cytologically confirmed NSCLC (squamous or non-squamous) without EGFR, ALK, or ROS1 alterations; with stage IIIb-IV disease according to the International Association for the Study of Lung Cancer (IASLC) Staging Manual in Thoracic Oncology (the 7th edition); with at least one measurable tumor lesion according to Response Evaluation for Criteria for Solid Tumors (RECIST) version 1.1; with Eastern Cooperative Oncology Group (ECOG) performance status of 0 or 1; and who had not taken antibiotics in the past three months. The key exclusion criteria were: untreated central nervous system metastases and patients previously taking anti-PD-1/PD-L1/PD-L2/CTLA-4 antibodies, antitumor vaccines, or systemic corticosteroids or immunosuppressants. All inclusion and exclusion criteria are available in the **Supplementary Appendix**.

Among the 168 patients accessed for eligibility, 108 subjects were excluded due to ineligibility (n = 90) or unwillingness to participate (n = 18). Finally, 60 patients met all the inclusion criteria and consented to participate (trial profile shown in **Supplementary Figure S1**). The Ethics Committee of the Chongqing University Cancer Hospital approved this study (CZLS2021042-A), registered at http://www.chictr.org.cn/(ChiCTR2200064690). The study design was not revised after the study began.

### 2.2 Study design and treatment

Patients were randomly (1:1) divided into two treatment groups using an interactive response technology system (1): Control group (n = 30): PD-1 plus chemotherapy plus placebo (2); JK5G group (n = 30): PD-1 plus chemotherapy plus JK5G postbiotics. Randomization was conducted using an interactive web response system with four blocks and stratified according to the histological classification (squamous vs. non-squamous), ECOG performance status score (0 vs. 1), cancer stage (IIIB/IIIC vs. IV), and gender (Male vs. Female). Patients and investigators were masked to the study treatment. The interactive web response system generated the allocation sequence, and the sponsor was masked to the allocation sequence. Masking was ensured by keeping JK5G and placebo identical in appearance, which were checked by a person responsible for investigational product allocation. Emergency code breaking via the interactive web response system was permitted during serious adverse events or by selecting other agents as later-line therapy. A combination of camrelizumab (200 mg), carboplatin (5 mg/mL per min, day 1), and pemetrexed (500 mg/m^2^, day 1) was used in non-squamous patients (23). Squamous patients received tislelizumab (200 mg) and carboplatin (AUC of 5), and paclitaxel (175 mg/m^2^) on day 1 of each of the four 3-week treatment cycles (24). Patients received 2.5 g of JK5G postbiotics or placebo three times a day before three meals of each of the four 3-week treatment cycles. Fecal samples were collected within 24 h before the treatment and when the patients finished the four treatment cycles. The JK5G microecological preparation is a postbiotic with high concentrations of more than 21 inactivated *Lactobacillus* strains and their metabolites (17). JK5G was purchased from JAPAN KYOWA INDUSTRIAL CO. LTD. (Tokyo, Japan). We representatively sequenced four groups of fecal samples: Control group before treatment (C0); Control group after four treatment cycles (C4); JK5G group before treatment (J0); JK5G group after four treatment cycles (J4).

All patients were instructed to consume during the four cycles following dietary guidelines and to engage in regular physical activities. Using the 2021 Guidelines of the Chinese Society of Clinical Oncology Nutrition in cancer patients, we calculated dietary calorific intakes based on the initial basal energy expenditure and physical activity levels (25). The weekly recipe was developed using the 2016 Dietary Guidelines for Chinese Residents (26).

After enrollment in both groups, health-related quality of life (QoL), nutrition level, pain, and psychological assessments were conducted every cycle. The QoL was evaluated by the European Organization for Reasearch and Treatment of Cancer Quality-of-Life Questionnaire Core 30 (EORCT QLQ-C30) version 3.0 and the Functional Assessment of Cancer Therapy-Lung (FACT-L) scale, comprising a multidimensional analysis of the QoL (function and symptom) during the previous week (27). Nutritional evaluation was performed using Patient-Generated Subjective Global Assessment (PG-SGA) (27). The Numerical Rating Scale (NRS) was used to assess pain (27). The Hospital Anxiety and Depression Scale (HADS), which evaluates anxiety (HADS-A) and depression (HADS-D) symptoms, as well as the Patient Health Questionnaire-9 (PHQ-9), were used for psychological evaluations in the previous week (27).

### 2.3 Outcomes

The primary endpoint was the objective response rate. The secondary endpoints were QoL, adverse effects incidence rate, 16S DNA sequencing of gut microbiota, blood inflammatory cytokines, and lymphocyte subsets.

A safety assessment was conducted once every 3-weeks during treatment to monitor adverse events and abnormal lab results. To categorize adverse events, we used the Medical Dictionary for Regulatory Activities (v. 21.0) and rated them according to the Common Terminology Criteria for Adverse Events (v. 5.0) of the National Cancer Institute.

### 2.4 Enzyme-linked immuno sorbent assay and flow cytometry

The serum levels of TNF-α, IL-2, IL-6, IL-8, and CRP were measured using ELISA kits, along with prealbumin (PA) and albumin (ALB) levels (R&D Systems, USA). We analyzed T lymphocyte subsets using ten-color flow cytometry (BD FACS Canto II) based on the lyse/no-wash procedure as previously described (28). All measurements were performed at least three times.

### 2.5 16S DNA gene sequencing

Fecal DNA samples were obtained, amplified, quantified, and sequenced on an Illumina PE250 platform (Illumina, San Diego, USA). Principal Coordinates Analysis (PCoA), linear discriminant analysis effect size (LEfSe), and the Canonical correlation analysis (CCA) were performed using Vegan v2.4.3 package (29). The LEfSe test distinguishes groups of microbes based on their characteristic features. The correlation between bacterial community structure and environmental factors was investigated using the CCA test (29, 30). See the **Supplementary Appendix** for more information.

### 2.6 Microbial function prediction and enterotyping

The Phylogenetic Investigation of Communities by Reconstruction of Unobserved States (PICRUSt) (version 2.2.0) was used to predict microbial function (29). The genes and functions predicted were aligned with the KEGG database using QIIME (version 1.9.1) and STAMP (*p* of 0.05 and effect size of 1). To cluster the gut enterotypes, we calculated the unweighted UniFrac distances based on the relative abundance between genera using R (v.3.2.2) as previously described (31). See the **Supplementary Appendix** for more information.

### 2.7 Analysis of short-chain fatty acids

The analysis of SCFAs, including acetate (338826; Sigma-Aldrich), propionate (402907; Sigma-Aldrich), and butyrate (19215; Sigma-Aldrich), was conducted using the Agilent 6890N GC system (Agilent Technologies, PA, USA) as previously described. The 2-thylbutyric acid (109959; Sigma-Aldrich) was used as an internal reference standard (29). Briefly, fecal pellets from each patient were weighted and homogenized in 1 mL of deionized water for 3 min. The pH of the suspension was adjusted to 2-3, which was subsequently transferred to a polypropylene tube and centrifuged for 20 min at 3,000g, yielding a clear supernatant. The 2-ethylbutyric acid (TEBA) was used as the internal standard and added to the supernatant at a final concentration of 1 mM.

### 2.8 Statistical analysis

We determined that a sample of 54 subjects would provide this study with 80% statistical power to detect an 8-point mean difference between groups in the FACT-L score with a two-sided significance level of 5% (27). Then, we considered a 10% loss of follow-up rate for the enrollment of an additional six participants to compensate for the loss of any patients to follow-up. Finally, 60 patients were enrolled. Data analysis was conducted using SPSS 24.0. Descriptive statistics were applied to estimate frequencies, means, and standard deviations. Fisher’s exact and χ2 tests were used for categorical variables to assess differences between groups for baseline characteristics and clinical outcomes. Independent-sample Student’s t-tests were used for continuous variables. The effect size was determined using Cohen’s d statistic, which measures the difference between two means divided by a standard deviation for the pooled data. According to the conventional classification, an effect size of 0.20 is small, 0.50 moderate, and 0.80 large. A *p* < 0.05 was considered statistically significant: *p < 0.05, **p < 0.01, ***p < 0.001; ns, no significance.

## 3 Results

### 3.1 Baseline characteristics of patients

Sixty patients were enrolled, received the allocated treatments, and were included in all analyses and safety sets: 30 received camrelizumab or tislelizumab plus chemotherapy plus placebo (control group), and 30 received camrelizumab or tislelizumab plus chemotherapy plus JK5G (JK5G group). The baseline characteristics were similar for both groups (**Table 1**). Patients were matched on age, sex, height, weight, BMI, ECOG, smoking status, histology, and cancer stage. The groups did not differ regarding the baseline nutrition assessment, pain score, mood symptoms, or QoL (**Table 1**).

**Table 1:** Demographic and baseline characteristics of the patients.

Characteristic	Control group (n=30)	JK-5G group (n=30)	*t/*χ^2^ */Z*	*P*
**Age, years**	61.10 ± 9.51	61.07 ± 8.67	0.01	0.989^a^
**Sex—no.(%)**			0.22	0.640^b^
Male	28 (93.33%)	27 (90.00%)		
Female	2 (6.67%)	3 (10.00%)		
**Height,cm**	165.73 ± 7.33	163.03 ± 6.88	1.47	0.147^a^
**Weight, kg**	62.57 ± 9.69	61.20 ± 8.01	0.60	0.552^a^
**BMI, kg/m 2**	22.35 ± 2.29	22.98 ± 2.68	-0.97	0.337^a^
**ECOG—no.(%)**			1.76	0.184^b^
0	16 (53.33%)	21 (70.00%)		
1	14 (46.67%)	9 (30.00%)		
**Smoking status—no.(%)**			0.27	0.605^b^
Former	13 (43.33%)	15 (50.00%)		
Never	17 (56.67%)	15 (50.00%)		
**Histology—no.(%)**			0.2	0.602^b^
Adenocarcinoma	18 (60.00%)	16 (53.33%)		
Squamous cell	12 (40.00%)	14 (46.67%)		
**Cancer stage—no.(%)**			1.96	0.353^b^
IIIB/IIIC	26 (86.67%)	29 (96.67%)		
IV	4 (13.33%)	1 (3.33%)		
**PG-SGA score—no.(%)**			4.35	0.114^b^
0–1	5 (16.67%)	5 (16.67%)		
2–8	25 (83.33%)	21 (70.00%)		
≥9	0	4 (13.33%)		
**NRS score—no.(%)**			1.29	0.525^b^
No pain (0)	7 (23.33%)	11 (36.67%)		
Mild pain (1-3)	15 (50.00%)	12 (40.00%)		
Moderate pain (4–6)	8 (26.67%)	7 (23.33%)		
Severe pain (7-10)	0	0		
Assessment of mood symptoms				
HADS				
Anxiety subscale (HADS-A)	3.33 ± 2.26	2.47 ± 2.69	1.35	0.182^a^
Depression subscale (HADS-D)	2.33 ± 2.75	2.53 ± 2.66	-0.29	0.776^a^
**PHQ-9** Depression severity			1.36	0.243^b^
No (0-4)	20 (66.67%)	24 (80.00%)		
Mild (5-9)	10 (33.33%)	6 (20.00%)		
Moderate (10-14)	0	0		
Scores on quality-of-life measures				
FACT-L scale	110.37 ± 11.17	107.07 ± 12.28	1.09	0.281^a^
Lung-cancer subscale	31.57 ± 2.34	31.23 ± 2.50	0.53	0.596^a^
Trial Outcome Index	72.47 ± 6.66	69.80 ± 8.37	1.37	0.177^a^
EORCT QLQ-C30				
Functional Scales				
Physical Functioning	74.22 ± 22.20	77.56 ± 21.12	-0.60	0.554^a^
Role Functioning	78.89 ± 18.01	72.22 ± 18.74	1.41	0.165^a^
Emotional Functioning	86.11 ± 11.23	82.78 ± 12.56	1.08	0.283^a^
Cognitive Functioning	90.00 ± 10.36	90.56 ± 12.13	-0.19	0.849^a^
Social Functioning	82.22 ± 16.91	73.89 ± 19.42	1.77	0.082^a^
Global Health	69.44 ± 24.01	61.11 ± 21.14	1.43	0.159^a^
Symptom Scales				
Fatigue	21.85 ± 15.84	20.00 ± 17.36	0.43	0.668^a^
Nausea and Vomiting	5.56 ± 7.99	3.33 ± 6.78	1.16	0.25^a^
Pain	22.78 ± 16.07	25.00 ± 20.88	-0.46	0.646^a^
Dyspnoea	17.78 ± 16.91	13.33 ± 16.61	1.03	0.309^a^
Insomnia	32.22 ± 20.50	30.00 ± 29.49	0.34	0.736^a^
Appetite Loss	28.89 ± 22.72	22.22 ± 26.74	1.04	0.302^a^
Constipation	23.33 ± 31.75	14.44 ± 25.80	1.19	0.239^a^
Diarrhoea	0	0	0.00	1.000^a^
Financial Difficulties	14.44 ± 16.80	18.89 ± 20.87	-0.91	0.367^a^

Data are means ± SD or n (%). ECOG, Eastern Cooperative Oncology Group; PG-SGA, Patient-Generated Subjective Global Assessment; NRS, Numerical Rating Scale; HADS, Hospital Anxiety and Depression Scale; PHQ-9, Patient Health Questionnaire-9; FACT-L, Functional Assessment of Cancer Therapy-Lung; EORCT QLQ-C30, European O-rganization for Reasearch and Treatment of Cancer Quality-of-Life Questionnaire Core 30.

a p-values calculated with the independent-samples Student’s t-tests for continuous variables.

b p-values calculated with two-sided χ^2^ and Fisher’s exact tests for categorical variables.

### 3.2 Vital characteristics of patients after four treatment cycles

When the four cycles of treatment were completed, the nutrition assessment (PG-SGA) and mood symptoms (Depression subscale, HADS-D, and PHQ-9) significantly improved in the JK5G group compared to the control group (both *p* < 0.05; **Table 2**). JK5G treatment significantly affected the QoL, improving not only the FACT-L scale (115.63 ± 11.30 vs. 122.57 ± 4.52; *p* = 0.003) but also the trial outcome index (74.43 ± 8.15 vs. 78.87 ± 3.21; *p* =-0.007), role functioning (82.78 ± 25.70 vs. 100 ± 0; *p* = 0.001), emotional functioning (90.00 ± 12.26 vs. 96.95 ± 4.08; *p* = 0.005), and social functioning (82.78 ± 24.95 vs. 97.78 ± 8.46; *p* = 0.003) and reducing fatigue (9.26 ± 21.06 vs. 0; *p* = 0.019), pain (13.89 ± 20.57 vs. 1.67 ± 5.09; *p* = 0.003), insomnia (16.67 ± 25.90 vs. 2.22 ± 8.46; *p* = 0.005), appetite loss (11.11 ± 25.27 vs. 0; *p* = 0.019), and financial difficulties (27.78 ± 12.63 vs. 12.22 ± 16.34; *p* <0.01) compared to the control group (**Table 3**). The comparison of measures of QoL after the four treatment cycles showed that the JK5G group had significantly higher scores than the control group, including the total FACT-L scale, the QLQ-C30 role functioning, social functioning and reducing the pain and financial difficulties, with effect sizes in the large range (**Table 3**). These results indicated that NSCLC patients might benefit from JK5G postbiotics treatment by improving nutrition status, depression symptoms, and QoL.

**Table 2:** Analyses of patients’ characteristics after four treatment cycles.

Characteristic	Control group (n=30)	JK-5G group (n=30)	*t/*χ^2^ */Z*	*P*
**PG-SGA score—no.(%)**			11.05	0.004^b^
0–1	13 (43.33%)	25 (83.33%)		
2–8	14 (46.67%)	5 (16.67%)		
≥9	3 (10.00%)	0		
**NRS score—no.(%)**			3.07	0.143^b^
No pain (0)	19 (63.33%)	25 (83.33%)		
Mild pain (1-3)	11 (36.67%)	5 (16.67%)		
Moderate pain (4–6)	0	0		
Severe pain (7-10)	0	0		
Assessment of mood symptoms				
HADS				
Anxiety subscale (HADS-A)	1.70±1.60	1.03±0.89	1.99	0.052^a^
Depression subscale (HADS-D)	1.47±1.74	0.20**±0.61**	3.77	<0.01^a^
**PHQ-9** Depression severity			7.93	0.005^b^
No (0-4)	23 (76.67%)	30 (100%)		
Mild (5-9)	7 (23.33%)	0 (0%)		
Moderate(10-14)	0	0		

Data are means ±SD or n (%). PG-SGA, Patient-Generated Subjective Global Assessment; NRS, Numerical Rating Scale; HADS, Hospital Anxiety and Depression Scale; PHQ-9, Patient Health Questionnaire-9.

a p-values calculated with the independent-samples Student’s t-tests for continuous variables.

b p-values calculated with two-sided χ^2^ and Fisher’s exact tests for categorical variables.

**Table 3:** Analyses of quality-of-life outcomes after four treatment cycles.

Variable	Control group (n=30)	JK-5G group (n=30)	Difference between JK-5G and Control group (95% CI)	*P* ^a^	Effect Size ^c^
Scores on quality-of-life measures					
FACT-L scale	115.63 ± 11.30	122.57 ± 4.52	6.93(2.43 – 11.43)	0.003	0.805
Lung-cancer subscale	33.60 ± 1.22	33.70 ± 1.71	0.10(-0.67 – 0.87)	0.795	0.067
Trial Outcome Index	74.43 ± 8.15	78.87 ± 3.21	4.43(1.19 –7.67)	0.007	0.715
EORCT QLQ-C30					
Functional Scales					
Physical Functioning	85.11 ± 21.06	92.45 ± 6.00	7.47(-0.63 – 15.57)	0.071	0.484
Role Functioning	82.78 ± 25.70	100 ± 0	17.23(7.61 – 26.86)	0.001	0.945
Emotional Functioning	90.00 ± 12.26	96.95 ± 4.08	7.00(2.25 – 11.75)	0.005	0.773
Cognitive Functioning	97.22 ± 6.32	98.89 ± 4.23	1.70(-1.14 – 4.54)	0.235	0.301
Social Functioning	82.78 ± 24.95	97.78 ± 8.46	15.10(5.32 – 24.88)	0.003	0.809
Global Health	56.67 ± 14.91	52.78 ± 29.06	-3.97(-16.05 – 8.11)	0.517	0.171
Symptom Scales					
Fatigue	9.26 ± 21.06	0	-9.33(-17.26 – -1.41)	0.019	0.622
Nausea and Vomiting	0	0	0	1	0
Pain	13.89 ± 20.57	1.67 ± 5.09	-12.13(-19.99 – -4.27)	0.003	0.811
Dyspnoea	3.33 ± 10.17	4.44 ± 11.52	1.10(-4.46 – 6.66)	0.694	0.102
Insomnia	16.67 ± 25.90	2.22 ± 8.46	-14.47(-24.58 – -4.35)	0.005	0.75
Appetite Loss	11.11 ± 25.27	0	-11.17(-20.65 – -1.68)	0.019	0.622
Constipation	0.00	0.00	0	1	0
Diarrhoea	0.00	0.00	0	1	0
Financial Difficulties	27.78 ± 12.63	12.22 ± 16.34	-15.40(-22.88 – -7.92)	<0.01	1.065

Data are presented as means ± SD. CI, confidence interval; FACT-L, Functional Assessment of Cancer Therapy-Lung; EORCT QLQ-C30, European Organization for Reasearch and Treatment of Cancer Quality-of-Life Questionnaire Core 3.

a p-values calculated with the independent-samples Student’s t-tests for continuous variables.

c Cohen’s d statistic was used to determine effect size, a measure of the difference between two means divided by the standard deviation (in this case, the mean in the JK5G group minus the mean in the control group) divided by a standard deviation for the pooled data. As described in conventional classification, effects bigger than 0.20 are small, those bigger than 0.50 are moderate, and those bigger than 0.80 are large.

### 3.3 Efficacy and safety

The objective response rate was 36.67% (11/30) in the control group compared to 50.00% (15/30) in the JK5G group (*p* = 0.297). The most frequent treatment-related adverse events in the control group were anemia, decreased white blood cell count, alopecia, decreased appetite, and reactive cutaneous capillary endothelial proliferation (RCCEP) (**Table 4**). The treatment-related adverse events of grade 3 or worse showed in at least 10% of patients in the control group were asthenia, increased alanine aminotransferase, and increased aspartate aminotransferase (**Table 4**). Patients in the JK5G group had a lower incidence of anemia (63.33% vs. 13.33%, *p* < 0.001), decreased lymphocyte count (20.00% vs. 0%, *p* = 0.010), decreased appetite (53.33% vs. 16.67%, *p* = 0.003), nausea (33.33% vs. 6.67%, *p* = 0.010), and asthenia (30.00% vs. 6.67%, *p* = 0.017) than the control group (**Table 4**). Altogether, these results suggested that JK5G postbiotics might attenuate PD-1 treatment-related adverse events.

**Table 4:** Treatment-Related Adverse Events.

Adverse Events	Control group (n=30)		JK-5G group (n=30)		χ^2^	*P* ^b^
	Any Grade	Grade≥ 3	Any Grade	Grade≥ 3		
Hematological toxicities						
Anemia	19 (63.33%)	0	4 (13.33%)	0	15.86	0.001
White blood cell count decreased	18 (60.00%)	3 (10.00%)	11 (36.67%)	0	3.27	0.071
Neutrophil count decreased	9 (30.00%)	0	6 (20.00%)	0	0.80	0.371
Lymphocyte count decreased	6 (20.00%)	0	0	0	6.67	0.010
Platelet count decreased	5 (16.70%)	0	3 (10.00%)	0	0.58	0.706
Nonhematological toxicities						
Alopecia	21 (70.00%)	0	17 (56.67%)	0	1.15	0.284
Decreased appetite	16 (53.33%)	0	5 (16.67%)	0	8.86	0.003
RCCEP	16 (53.33%)	0	13 (43.33%)	0	0.60	0.438
Alanine aminotransferase increased	11 (36.67%)	3 (10.00%)	5 (16.67%)	0	3.07	0.080
Aspartate aminotransferase increased	11 (36.67%)	3 (10.00%)	5 (16.67%)	0	3.07	0.080
Nausea	10 (33.33%)	0	2 (6.67%)	0	6.67	0.010
Asthenia	9 (30.00%)	2 (6.67%)	2 (6.67%)	0	8.18	0.017
Pain in extremity	9 (30.00%))	2 (6.67%)	6 (20.00%)	0	0.80	0.371
Gamma-glutamyl transferase increased	6 (20.00%)	0	2 (6.67%)	0	2.40	0.254
Rash	5 (16.67%)	0	1 (3.33%)	0	3.21	0.195
Vomiting	4 (13.33%)	0	0	0	5.83	0.112
Arthralgia	3 (10.00%)	0	0	0	4.32	0.237
Hypoaesthesia	2 (6.67%)	0	2 (6.67%)	0	0.00	1.000
Hypothyroidism	1 (3.33%)	0	0	0	1.40	1.000
Pneumonia	1 (3.33%)	0	0	0	1.40	1.000

Data are presented in n (%). RCCEP, Reactive cutaneous capillary endothelial proliferation.

b p-values calculated with two-sided χ^2^ and Fisher’s exact tests for categorical variables.

### 3.4 JK5G postbiotics remodeled gut microbial communities and butyrate concentration

**Figure 1:**
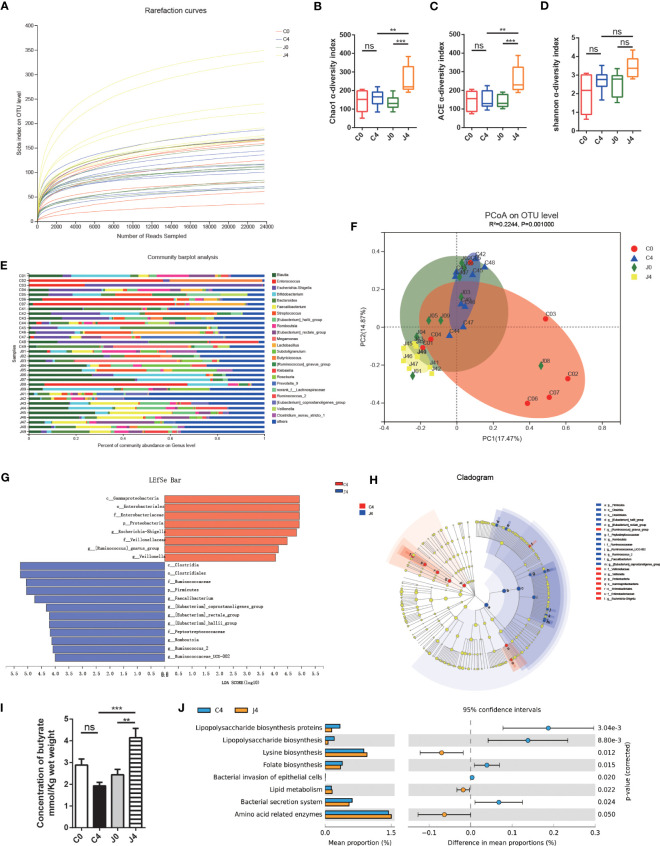
JK5G postbiotics remodeled gut microbial communities and butyrate concentration. **(A)** Rarefaction curves of sequencing samples at baseline and after four treatment cycles grouped by intervention status. The α diversity of intestinal microbial compositions evaluated by Chao1 **(B)**, ACE **(C)**, and Shannon **(D)** indexes at baseline and after four treatment cycles. Data are represented as medians with interquartile ranges. **(E)** Changes in the taxonomic composition of gut microbiota at baseline and after four treatment cycles. Stacked bar charts show the individual variability of the relative abundances of major bacterial genera. **(F)** Principal coordinate analysis (PCoA) based on an Bray-curtis distance matrix at the OTU level. **(G)** Linear Discriminant Analysis (LDA) scores derived from LEfSe showing the biomarker taxa (LDA score > 2 and *p* < 0.05 determined by the Wilcoxon signed-rank test). **(H)** Cladogram plots of gut microbiota in the C4 vs. J4 groups. **(I)** Butyrate concentration in fecal samples. **(J)** Function prediction of microbial genes involved in metabolism by PICRUSt analysis and Welch’s t-test (*p* < 0.05). For panel F, colorful circles represent 95% confidence intervals calculated by Welch’s inverted method. Data are presented as means ± SEM. **p < 0.01, ***p < 0.001, ns, no significance, compared to the marked groups. Multiple groups were tested by one-way ANOVA followed by Bonferroni analysis.

Moreover, we analyzed 1,517,002 sequences, and the rarefaction diversity results showed that most diversity was covered (**Figure 1A**). The Chao1 and abundance-based coverage estimation (ACE) index, measures of taxonomic α diversity, significantly differed during the study (**Figures 1B, C**). In contrast, the Shannon index did not differ (**Figure 1D**). After analyzing the gut microflora profile, we found a substantial variation in the bacterial community composition between samples (**Figure 1E**). According to Bray-curtis PCoA, JK5G treatment did not differ across study periods (**Figure 1F**). The gut microbiota of subjects remained relatively stable over time. The responses to JK5G postbiotics interventions were highly correlated with the initial gut microbiota state. Additionally, the LEfSe test and cladogram plots showed that the pernicious bacteria *Escherichia-Shigella* increased in the control group, while SCFA (e.g., butyrate)-producing bacteria, including *Faecalibacterium* and *Ruminococcaceae*, increased in the JK5G group after four treatment cycles (J4) (**Figures 1G, H**). Regarding the differences in the concentration of SCFAs in feces, the J4 group had a noticeable increase in butyrate compared to the control group after four treatment cycles (C4) (**Figure 1I**). Nevertheless, fecal acetate and propionate levels did not differ between the two groups (**Supplementary Figures S2A, B**). Then, we evaluated the functional changes associated with gut microbiota composition between groups using PICRUSt. In the J4 group, “lipopolysaccharide (LPS) biosynthesis proteins”, “lipopolysaccharide biosynthesis”, and “bacterial secretion system” pathways were downregulated compared to the C4 group (**Figure 1J**). On the other hand, the biomarkers for “Lysine biosynthesis”, “Lipid metabolism”, and “Amino acid related enzymes” pathways were greatly higher in the J4 group than in the C4 group (**Figure 1J**). These data suggested that JK5G postbiotics could restructure the gut microbiota of NSCLC patients, resulting in increased SCFA concentration, which might be related to regulating the tumor microenvironment.

**Figure 2:**
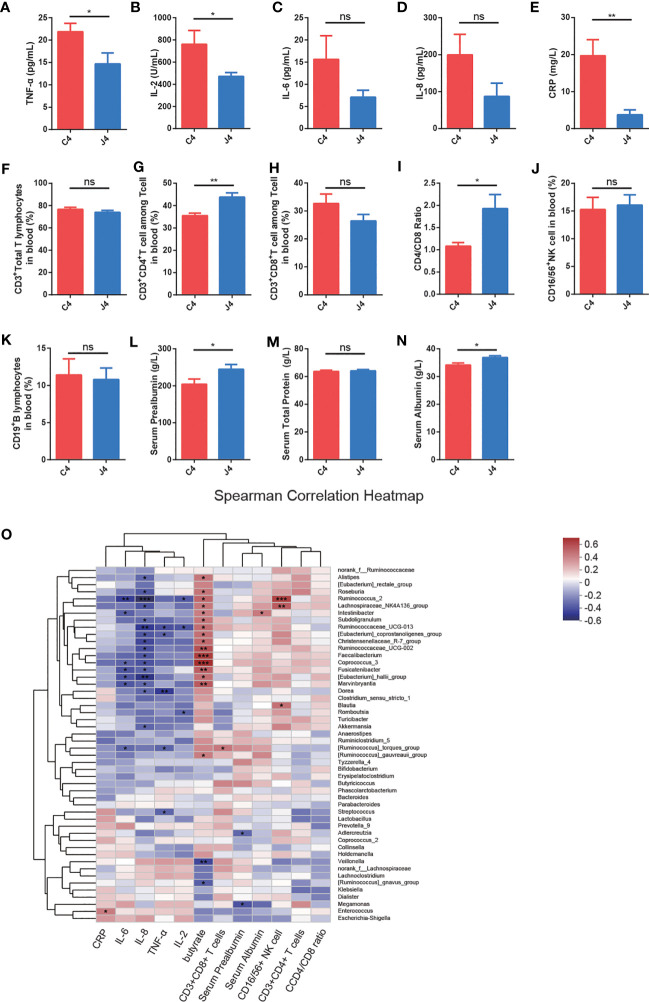
JK5G postbiotics ameliorate serum inflammatory cytokines, blood flow cytometry of lymphocyte subsets, and nutrition-related indicators. **(A-E)** TNF-α **(A)**, IL-2 **(B)**, IL-6 **(C)**, IL-8 **(D)**, and CRP **(E)** levels in patients between C4 and J4 groups. **(F-K)** The proportions of CD3^+^Total T lymphocytes **(F)**, CD3^+^CD4^+^T cells **(G)**, CD3^+^CD8^+^T cells **(H)**, CD4/CD8 ratio **(I)**, CD16/56^+^NK cells **(J)**, as well as CD19^+^B lymphocytes **(K)** in peripheral blood in the J4 group were remarkably increased compared to the C4 group. **(L-N)** Nutrition-related indicators serum Prealbumin **(L)**, Serum Total Protein **(M)**, and Serum Albumin **(N)** in blood. Student’s t-test. **(O)** The heatmap shows Spearman correlation coefficients between the abundance of 50 genera and the clinical indices, including serum inflammatory cytokines, blood flow cytometry of immune cells, nutrition-related indicators, fecal butyrate concentration, and bacterial genera. Red represents positive correlations, blue negative correlations, and white no correlation. For panel **(A-N)**, data are presented as means ± SEM, were calculated with the Student’s t-tests. *p < 0.05, **p < 0.01, ***p < 0.001, ns, no significance.

### 3.5 JK5G postbiotics ameliorate serum inflammatory cytokines, blood flow cytometry of the immune cells, and nutrition-related indicators

Emerging evidence has shown that inflammatory cytokines and the function of T cell subsets play a fundamental role in response to PD-1 blockade in cancer (32). To explore whether JK5G postbiotics ameliorated inflammatory cytokines, immune function, and nutritional state, we analyzed the serum inflammatory cytokines (**Figures 2A-E**), peripheral blood flow cytometry of immune cells (**Figures 2F–K**), and nutrition-related indicators (**Figures 2L–N**). Serum inflammatory cytokines were evaluated using ELISA kits (**Figure 2**). JK5G administration was associated with the levels of pro-inflammatory markers, including TNF-α (**Figure 2A**), IL-2 (**Figure 2B**), and CRP (**Figure 2E**). Furthermore, we analyzed T (CD3^+^ Total T lymphocytes), CD3^+^CD4^+^ T, CD3^+^CD8^+^ T, natural killer (CD16/56^+^ NK cell), and B (CD19^+^ B lymphocytes) cells in peripheral blood (**Figures 2F–K**). The J4 group showed significant increases in CD3^+^CD4^+^ T cells (**Figure 2G**) and CD4/CD8 ratios (**Figure 2I**) compared to C4 (all *p* < 0.05). Additionally, compared to the C4 group, serum prealbumin (**Figure 2L**) and albumin (**Figure 2N**) levels were significantly higher in J4 (all *p* < 0.05). NSCLC patients presented significant positive correlations between *Faecalibacterium*, *Ruminococcaceae_UCG-002*, *Coprococcus_3*, and feces butyrate concentration (**Figure 2O**). A positive correlation was observed between *Ruminococcus, Lachnospiraceae_NK4A136_group*, and the percentage of CD16/56+NK cells in peripheral blood. Meanwhile, *Ruminococcus* and *Ruminococcaceae_UCG-013* were significantly negatively correlated with serum IL-2 and IL-8. These results demonstrated that JK5G postbiotic treatment regulated immune cells and attenuated inflammatory cytokines, which might further benefit the immune function of NSCLC patients.

### 3.6 Stratification and functional characteristics of enterotypes

**Figure 3:**
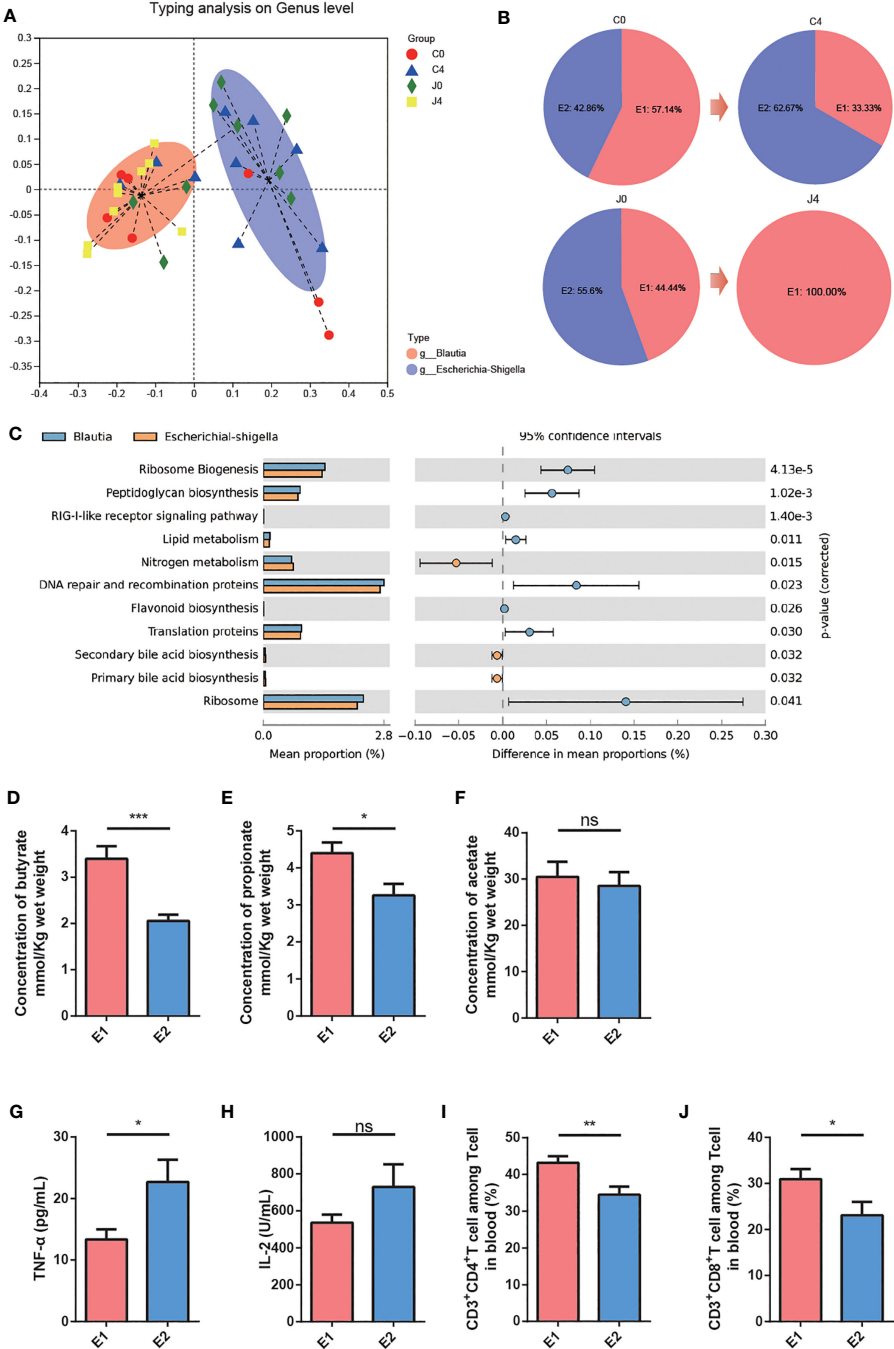
Enterotype stratification and functional characteristics. **(A)** The first two principal coordinates of unweighted UniFrac distances of the relative abundance profiles at the genus level. Samples are colored red for *Blautia* enterotype (E1) and blue for *Escherichia-Shigella* enterotype (E2). **(B)** Enterotype composition in the four groups. **(C)** Comparisons of relative gene abundances in KEGG reference pathways between E1 and E2 subjects. The bar graphs on the left display the mean proportion of each microbial taxon. Dot plots on the right display the difference in mean proportions between two enterotypes with associated *p*-values. Error bars of dots represent the 95% CI. **(D-F)** Fecal butyrate **(D)**, propionate **(E)**, and acetate **(F)** levels for E1 and E2 subjects. **(G, H)** Serum TNF-α **(G)** and IL-2 **(H)** levels for E1 and E2 subjects. **(I-J)** The proportions of CD3^+^CD4^+^T cells **(I)** and CD3^+^CD8^+^T cells **(J)** in the peripheral blood of E1 subjects were remarkably increased compared to E2. For panel **(D –J)**, data are presented as means ± SEM, and differences were analyzed with the independent-samples Student’s t-tests. *p < 0.05, **p < 0.01, ***p < 0.001, ns, no significance.

Many studies have suggested that enterotypes can contribute to the identification of disease states. Enterotyping might guide treatment options and help understand different treatment responses (19, 20). Thus, to explore the relationship between the gut microbiome and treatment response, we simultaneously clustered the gut microbial enterotypes among NSCLC patients using unweighted UniFrac distance metrics (**Figure 3A**). Two enterotypes were identified: *Blautia* (E1) and *Escherichia-Shigella* (E2), driven by *Blautia* and *Escherichia-Shigella* levels, respectively (**Figure 3A**). Compared to J4 and J0, after JK5G postbiotics intervention, the E1 composition converted from 44.44 to 100.00% (**Figure 3B**). To evaluate the functional changes associated with gut microbiota composition, we used PICRUSt and found that the gut microbiota function greatly differed between E1 and E2 groups. A higher gene activity of “ribosome biogenesis”, “peptidoglycan biosynthesis,” and “lipid metabolism” was detected in the E1 group. Meanwhile, a higher gene activity of “nitrogen metabolism” was observed in the E2 group (**Figure 3C**).

Furthermore, we identified the differences in essential parameters within and between enterotypes during the study periods. E1 subjects had significantly improved fecal butyrate (**Figure 3D**) and propionate (**Figure 3E**) levels than E2 subjects. Nevertheless, fecal acetate levels did not differ between the two enterotype groups (**Figure 3F**). And E1 subjects had decreased plasma TNF-α (**Figure 3G**) levels than E2 subjects. There was no difference in Plasma IL-2 between the two groups (**Figure 3H**). Additionally, the proportions of CD3^+^CD4^+^T cells (**Figure 3I**) and CD3^+^CD8^+^T cells (**Figure 3J**) in peripheral blood in the E1 group remarkably increased compared to the E2 group. These results indicated that the benefits of JK5G postbiotics intervention might be attributed to enterotype modulations.

### 3.7 Associations between samples, microbial taxons, and physiological biochemistry factors by canonical correlation analysis

**Figure 4:**
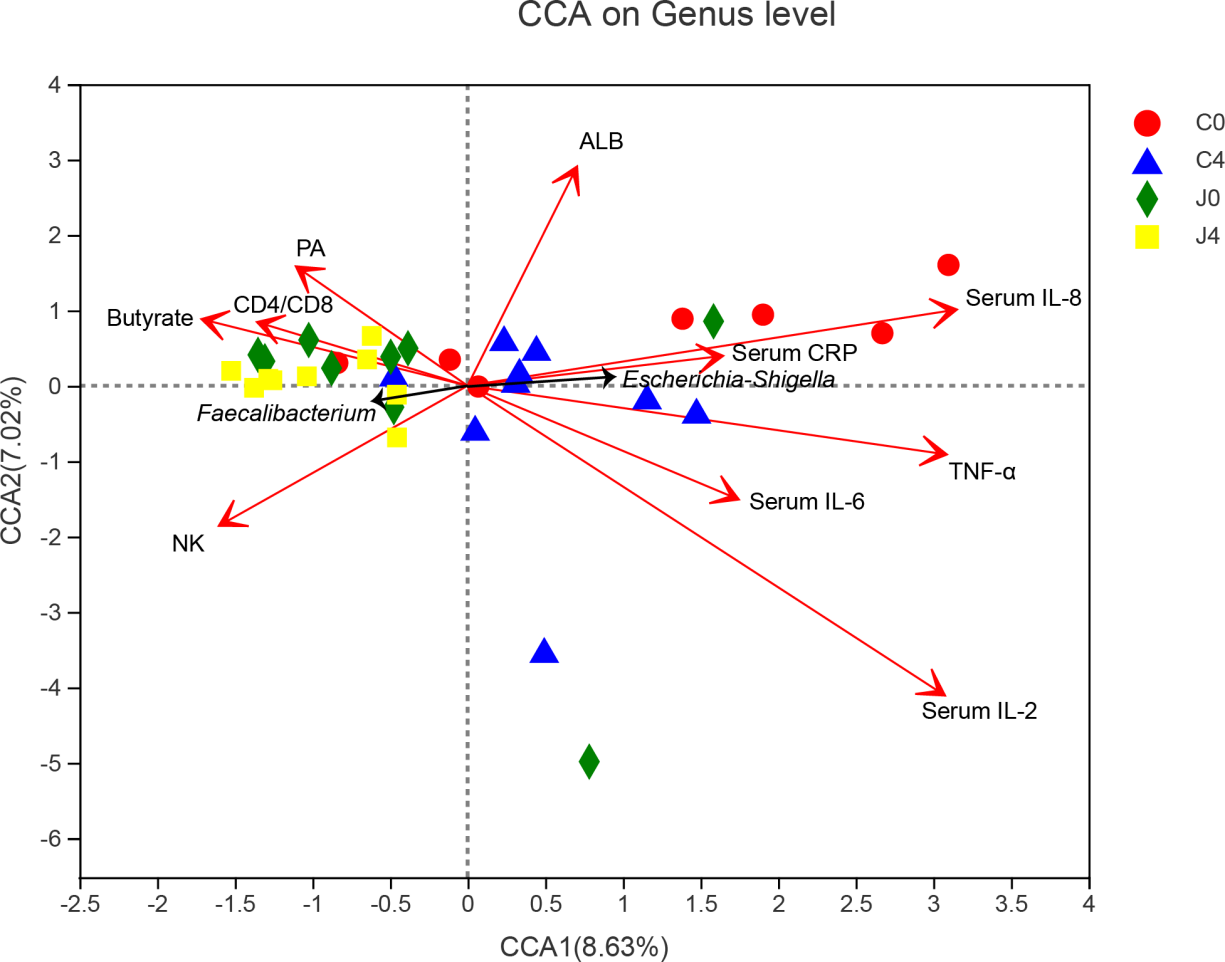
Canonical correlation analysis (CCA) diagram among samples, microbial species, and physiological biochemistry factors. Characteristic bacteria (black arrows), samples (symbols), and physiological biochemistry factors (red arrows) are shown in the diagram. The values of axes 1 and 2 are the percentages explained by the corresponding axis. Names of parameters are drawn as vectors by their association with the first two components.

We used CCA to establish the potential correlation between samples, bacterial communities, and physiological biochemistry factors (**Figure 4**). In the physiological biochemistry factors arrows, the length represents the influence of those factors on bacteria data, while the angle represents the correlation. Regarding the angle formed between arrows, an acute angle means a positive correlation, an obtuse angle means a negative correlation and a right angle shows no correlation. *Faecalibacterium* was positively correlated to the fecal butyrate concentration, CD4/CD8 ratio, and serum prealbumin (PA). Pro-inflammatory cytokines, including TNF-α, IL-2, IL-6, IL-8, and CRP, were significantly correlated with characteristic *Escherichia-Shigella* bacteria.

## 4 Discussion

In the present study, we showed that treatment with JK5G postbiotics was associated with favorable QoL and clinical indexes and a lower rate of irAEs in advanced NSCLC patients under ICIs plus chemotherapy. To our knowledge, this is the first trial to report the effects of JK5G postbiotics on the treatment efficacy and irAEs in advanced NSCLC patients under ICIs plus chemotherapy. Given the progressive nature of NSCLC, improving the QoL, nutrition status, and psychological well-being is challenging (27). Here, patients treated with JK5G presented improved FACT-L and EORCT QLQ-C30 scales, represented by increased role functioning, emotional functioning, and social functioning, and reduced fatigue, pain, insomnia, appetite loss, and financial difficulties in the J4 group compared to the C4 group. These groups also differed in depression scores.

Most irAEs were of grade 1 or 2. Commonly reported irAEs included hematological and nonhematological toxicities, such as anemia, decreased white blood cell count, alopecia, decreased appetite, and nausea, consistent with previous studies (4, 24). Patients in the JK5G group had a lower incidence of anemia, decreased lymphocyte count, decreased appetite, nausea, and asthenia than the control group. These results suggested that JK5G might attenuate PD-1 treatment-related adverse events, improving the QoL and nutritional level of NSCLC patients.

When the microecology combines with intestinal mucosa, the intestinal tract mucosa is strengthened with a greater barrier and anti-bacterial function, pathogenic bacteria are inhibited, the microbiota is distributed better, and immunity is enhanced under postbiotics or beneficial bacteria supplementation (33). Postbiotics are a potential novel therapeutic strategy to modulate microbiome and immunotherapy in cancer patients (11). Usually, NSCLC patients are accompanied by elevated TNF-α, IL-2, and CRP levels (34) and tend to have a reduction in Tregs, lymphocytes, T cells, and CD3^+^CD4^+^ T cells (34). An animals study reported that JK5G could slow the development of colorectal cancer and inhibit inflammatory factors in serum while the spleen became more densely loaded with lymphocytes, such as T, CD3^+^CD4^+^ T, and CD3^+^CD8^+^ T cells (17). Similarly, we found that JK5G postbiotics treatment might decrease pro-inflammatory markers, including TNF-α, IL-2, and CRP. Moreover, the J4 group showed significant increases in CD3^+^CD4^+^ T cells and CD4/CD8 ratio compared to the C4 group. JK5G treatment also improved nutrition-related indicators, including serum prealbumin and albumin. These data suggested that JK5G treatment can improve immune function and nutritional status and reduce the inflammatory response in NSCLC patients under ICIs.

The JK5G postbiotics is a high-concentration complex rich in bacteria and their metabolites, including *Lactococcus* and 21 *Lactobacillus* and peptidoglycans and metabolites from *Lactococcus* cytoderm. The most important components of JK5G are SCFAs and inactivated *Lactobacillus*, which can directly reach the intestines without being affected by stomach acid and bile. Recent studies have found that dietary *Lactobacillus* exopolysaccharides induce CCR6^+^ CD8^+^ T cells in Peyer’s patches and enhance the therapeutic effects of the immune-checkpoint blockade (35). Moreover, *Lactobacillus rhamnosus* Probio-M9 can enhance the effects of anti-PD-1 antitumor therapy by restoring antibiotic-disrupted gut microbiota (33). Our results showed that after the treatment with JK5G postbiotics, butyrate-producing *Faecalibacterium* and *Ruminococcaceae* were more abundant in NSCLC patients. Furthermore, the JK5G group had a noticeable increase in feces butyrate concentration. Previous studies have emphasized that butyrate might enhance anticancer efficacy in the CD8^+^ T cell immunity pathway, modulate immune responses, and improve adoptive immunotherapy for cancer (36, 37). Butyrate also stimulates Treg cells, resulting in better intestinal barrier function since it suppresses proinflammatory cytokines (30). Altogether, these results demonstrated that the beneficial effects of JK5G might be related to increased butyrate-producing bacteria and enhanced fecal butyrate concentration, which might be further involved in regulating the T cell immunity pathway and immune responses. Hence, JK5G postbiotics can regulate the tumor microenvironment via the gut microbiota.

The relative abundance of *Ruminococcaceae* and *Faecalibacterium* significantly increases in ICIs-responding patients (3), which usually implies a better clinical outcome. Similar to our results, *Ruminococcaceae* and *Faecalibacterium* increased after treatment with JK5G postbiotics, accompanied by a better QoL and nutritional status. Thus, by improving T cell function in the peripheral blood and tumor microenvironment, *Ruminococcaceae* and *Faecalibacterium* can enhance systemic and anti-tumor immune responses.

Emerging evidence has shown that enterotypes can help identify disease states and understand different treatment responses (19, 20). Colorectal cancer patients have been identified with enterotypes including *Alistipes*, *Bacteroides*, and *Prevotella* (21). However, the enterotypes of lung cancer patients have not been explored. Herein, we identified two enterotypes in NSCLC patients: *Blautia* (E1) and *Escherichia-Shigella* (E2). After the JK5G intervention, the E1 composition converted from 44.44 to 100.00% in the J4 group. E1 subjects had significantly improved fecal butyrate and propionate and decreased inflammatory factors than E2 subjects. Furthermore, a significant increase in CD3^+^CD4^+^T and CD3^+^CD8^+^T cells was observed in the peripheral blood of E1 subjects. A report suggested that *Blautia* is enriched in ICIs-responding NSCLC patients and mice and correlated with decreased tumor growth in the immunotherapy response area (38). Therefore, patients clustered into *Blautia* enterotypes (E1) might be more responsive to immunotherapy. However, few studies have been conducted on gut enterotypes in cancer. Thus, the biological and clinical significance of enterotypes needs to be further explored. Our data supported that JK5G postbiotics might influence the patient’s response to ICIs by changing the gut microbiota and enterotypes.

However, our current study also has some limitations. First, the sample size was relatively small, and this study was conducted in only one institution with Chinese patients, making it difficult to generalize the results to other populations or settings. To minimize this influence, numerous measures were taken to ensure data validity, including an interactive response technology system and a double-masked, placebo-controlled trial method. Moreover, to limit the influence of food and lifestyles on subjects, all patients were instructed to consume during the four cycles following dietary guidelines and to engage in regular physical activities. Second, the follow-up period was short, and no survival data was collected. We planned to conduct a larger-scale multicenter study and longer follow-up times in the future. Third, 16S rRNA sequencing might not illustrate the whole gut microbiota signature, whereas metagenomics sequencing would provide a more comprehensive analysis. Finally, the role of JK5G in regulating the structure and function of the intestinal flora in NSCLC patients under ICIs remains unclear. Additionally, via which pathways JK5G postbiotics regulate the gut microbiota needs to be further verified.

## 5 Conclusion

In summary, supplementation with JK5G postbiotics might attenuate the incidence rate of irAEs and enhance the quality of life and nutritional status in advanced NSCLC patients who received ICIs. Moreover, JK5G postbiotics could improve intestinal microbial structures and enterotypes. Hence, JK5G postbiotics might be an adjuvant for NSCLC patients expected to receive ICIs.

## Data availability statement

The datasets presented in this study can be found in online repositories. The names of the repository/repositories and accession number(s) can be found below: https://www.ncbi.nlm.nih.gov/, accession number PRJNA914067.

## Ethics statement

The Ethics Committee of the Chongqing University Cancer Hospital approved this study (CZLS2021042-A). The patients/participants provided their written informed consent to participate in this study.

## Author contributions

MC initiated the project, designed and performed experiments, analyzed the data and drafted the manuscript; MC, and LM, collected samples and performed the experiments. SH, JZ, JG, LY, LC, HL, LT, SW enrolled and followed up the patients. HY and MC designed the project, obtained funding, helped with the writing of the paper, and finalized the manuscript. All authors read and approved the final manuscript.

## Supplementary material

Supplementary Figure 2 Fecal acetate (A) and propionate (B) levels among different groups. Data are reported as means ± SEM; ns, no significance. Multiple groups were tested by one-way ANOVA followed by Bonferroni’s *post hoc* test.
