# Supplementary material for: JK5G postbiotics attenuate immune-related adverse events in NSCLC patients by regulating gut microbiota: a randomized controlled trial in China
Source: Front Oncol. 2023 Aug 4;13:1155592. doi: 10.3389/fonc.2023.1155592 (PMC10436471; doi:10.3389/fonc.2023.1155592)
Supplement: Supplementary file 1 [file DataSheet_1.doc]

**Supplementary Appendix.**

1. **Inclusion and exclusion criteria**

**1.1 Inclusion criteria**

(1) Patients with advanced non-small cell lung cancer diagnosed for the first time; had stage IIIb-IV disease according to International Association for the Study of Lung Cancer (IASLC) Staging Manual in Thoracic Oncology (the 7th edition), histologically or cytologically confirmed NSCLC (squamous or non-squamous) without EGFR, ALK, or ROS1 alterations

(2) Age ≥ 40 years old and ≤ 70 years old;

(3) ECOG score: 0 ~ 1 points;

(4) Expected survival period is more than 12 weeks;

(5) According to RECIST 1.1, at least one target lesion can be measured;

(6) The patient can eat by mouth normally;

(7) Had not taken antibiotics in the past 3 months, treatment-naive;

(8) The function of main organs is normal, that is, it meets the following standards:

(9) Good hematopoietic function, defined as white blood cell count ≥3.0 × 10^9^/L, absolute neutrophil count ≥1.5 × 10^9^/L, platelet count ≥100 × 10^9^/L, hemoglobin ≥90 g/L (without transfusion within 14 days).

(10) Biochemical examinations must meet the following standards: TBIL < 1.25 times the upper limit of normal value (ULN); ALT and AST < 2.5ULN; if there is liver metastasis, ALT and AST < 5ULN;

(11) Good renal function: Cr ≤ 1.5ULN or creatinine clearance (CCr) ≥50 ml/min;

(12) Good coagulation function, INR and APPT ≤ 1.5 times ULN; if the subject is receiving anticoagulant therapy, as long as APPT is within the intended use range of anticoagulant drugs;

(13) If Serum lipase were 1.5 x ULN (lipase > 1.5 ULN without clinical or radiographic confirmed pancreatitis can be enrollment); Serum amylase were 1.5 ULN (if amylase > 1.5 ULN without clinical or radiographic confirmed pancreatitis can be enrollment); Alkaline phosphatase(ALP) ≤2.5 ULN, if there is liver metastasis, ALP≤2.5 ULN.

(14) Women of childbearing age should agree to use contraceptive measures (such as an IUD, birth control pills or condoms) during the study period and within 6 months after the end of the study; Serum or urine pregnancy test is negative within 7 days before study enrollment, and must be a non-lactating patient; Men should agree to patients who must use contraception during the study period and within 6 months after the end of the study period.

(15) Patients voluntarily joined the study, signed informed consent, and had good compliance.

**1.2 Exclusion criteria**

(1) Can't eat through the mouth;

(2) Ever received any t-cell stimulation or immune checkpoint treatment. Including but not limited to cytotoxic T lymphocyte associated antigen 4 (CTLA-4) inhibitors, PD - L1 inhibitors, PD - 1/2 inhibitors or other targeted drugs of T cells; Ever received antitumor vaccines.

(3) Known symptomatic brain metastasis, spinal cord compression, cancerous meningitis, or brain or pia mater disease detected by CT or MRI;

(4) Those with a history of psychotropic substance abuse who are unable to quit or have mental disorders;

(5) Patients participating in other clinical studies;

(6) Patients with autoimmune diseases or immunodeficiency diseases; had systemic corticosteroids or immunosuppressants;

(7) Known allogeneic organ transplantation (except corneal transplantation) or allogeneic hematopoietic stem cell transplantation.

(8) Patients with co-existence of other malignant tumors, except those that have been cured such as cervical carcinoma in situ and non-melanoma skin cancer, or concomitant diseases that according to the investigators’ judgment would seriously endanger the safety of the patient or affect the completion of the study.

**1.3 Early withdrawal/termination criteria**

(1) Patient who cannot tolerate 4 cycles of anti-PD-1 antibody and chemotherapy;

(2) Patients who are difficult to complete the baseline test;

(3) Patients who cannot take JK-5G regularly and on time;

(4) Patients with serious adverse events;

1. Patients with medical imaging evidence of significant progression or death;
2. Patients who violate the test plan;
3. Patients who voluntarily request to withdraw from the test.
4. **Supplemental Methods**

**2.1 DNA extraction and PCR amplification**

The samples were stored at -80 °C until genomic DNA (gDNA) extraction using the QIAamp DNA Stool Mini Kit (Qiagen, Hilden, Germany). The gDNA concentration and purity were estimated using a Nanodrop 1000 spectrophotometer. The hypervariable region V3-V4 of the bacterial 16S rRNA gene were amplified with primer pairs 338F (5'-ACTCCTACGGGAGGCAGCAG-3') and 806R(5'-GGACTACHVGGGTWTCTAAT-3') ^[1]^ by an ABI GeneAmp® 9700 PCR thermocycler (ABI, CA, USA). The PCR reaction mixture including 4 μL 5 × Fast Pfu buffer, 2 μL 2.5 mM dNTPs, 0.8 μL each primer (5 μM), 0.4 μL Fast Pfu polymerase, 10 ng of template DNA, and ddH_2_O to a final volume of 20 µL. PCR amplification cycling conditions were as follows: initial denaturation at 95 ℃ for 3 min, followed by 27 cycles of denaturing at 95 ℃ for 30 s, annealing at 55 ℃ for 30 s and extension at 72 ℃for 45 s, and single extension at 72 ℃ for 10 min, and end at 4 ℃. All samples were amplified in triplicate. The PCR product was extracted from 2% agarose gel and purified. Then quantified using Quantus™ Fluorometer (Promega, USA)

**2.2 Illumina sequencing**
Purified amplicons were pooled in equimolar amounts and paired-end sequenced on an Illumina PE250 platform (Illumina, San Diego,USA) according to the standard protocols by Majorbio Bio-Pharm Technology Co. Ltd. (Shanghai, China). The raw sequencing reads were deposited into the NCBI database (accession number PRJNA914067).

**2.3 Data processing**

Raw FASTQ files were de-multiplexed using an in-house perl script, and then quality-filtered by fastp version 0.19.6^[2]^ and merged by FLASH version 1.2.11^[3]^ with the following criteria:

(i) the 300 bp reads were truncated at any site receiving an average quality score of <20 over a 50 bp sliding window, and the truncated reads shorter than 50 bp were discarded, reads containing ambiguous characters were also discarded; (ii) only overlapping sequences longer than 10 bp were assembled according to their overlapped sequence. The maximum mismatch ratio of overlap region is 0.2. Reads that could not be assembled were discarded; (iii) Samples were distinguished according to the barcode and primers, and the sequence direction was adjusted, exact barcode matching, 2 nucleotide mismatch in primer matching. Then the optimized sequences were clustered into operational taxonomic units (OTUs) using UPARSE 11 ^[4,5]^ with 97% sequence similarity level. The most abundant sequence for each OTU was selected as a representative sequence. The OTU table was manually filtered, i.e., chloroplast sequences in all samples were removed. To minimize the effects of sequencing depth on alpha and beta diversity measure, the number of 16S rRNA gene sequences from each sample were rarefied to 20,000, which still yielded an average Good’s coverage of 99.09%, respectively.

The taxonomy of each OTU representative sequence was analyzed by RDP Classifier version 2.13^[6]^ against the 16S rRNA gene database Silva v138 using confidence threshold of 0.7. The metagenomic function was predicted by PICRUSt (Phylogenetic Investigation of Communities by Reconstruction of Unobserved States) software (version 1.1.0) ^[7]^ based on OTU representative sequences. The predicted genes and functions were aligned to KEGG database (version 66.1, May 1, 2013).

**2.4 Statistical Analysis**

Bioinformatic analysis of the gut microbiota was carried out using the Majorbio Cloud platform ([https://cloud.majorbio.com](https://cloud.majorbio.com/)). Based on the OTUs information, rarefaction curves and alpha diversity indices including observed OTUs, Chao1 richness, Abundance-based Coverage Estimator (ACE), Shannon index and Good’ s coverage were calculated with Mothur v1.30.2^[8]^. The similarity among the microbial communities in different samples was determined by principal coordinate analysis (PCoA) based on Bray-curtis distance matrix using Vegan v2.4.3 package. The PERMANOVA test was used to assess the percentage of variation explained by the treatment along with its statistical significance using Vegan v2.4.3 package. The linear discriminant analysis (LDA) effect size (LEfSe) ^[9]^ (http://huttenhower.sph.harvard.edu/LEfSe) was performed to identify the significantly abundant taxa (phylum to genera) of bacteria among the different groups (LDA score > 2, P < 0.05). STAMP (version 2.0.9) was utilized to determine significant putative KEGG orthologs and pathway analyses with two-side Welch’s t-test including two filters (*P* value < 0.05 and effect size > 0.2) applied to present features ^[10]^. A correlation between two nodes was considered to be statistically robust if the spearman’s correlation coefficient over 0.6 or less than -0.6, and the P-value less than 0.05. The correlation between bacterial community structure and environmental factors was investigated using the Canonical correlation analysis (CCA) test ^[11,12]^.

**References**

[1] Liu C, Zhao D, Ma W, et al. Denitrifying sulfide removal process on high-salinity wastewaters in the presence of Halomonas sp[J]. Applied microbiology and biotechnology, 2016, 100(3): 1421-1426.

[2] Chen S, Zhou Y, Chen Y, et al. fastp: an ultra-fast all-in-one FASTQ preprocessor[J]. Bioinformatics, 2018, 34(17):i884-i890.

[3] Tanja, Mago, Steven, et al. FLASH: fast length adjustment of short reads to improve genome assemblies.[J]. Bioinformatics, 2011, 27(21):2957‐2963.

[4] Edgar, Robert C. UPARSE: highly accurate OTU sequences from microbial amplicon reads.[J]. Nature Methods, 2013, 10(10):996‐998.

[5] Stackebrandt E, Goebel B M . Taxonomic Note: A Place for DNA-DNA Reassociation and 16S rRNA Sequence Analysis in the Present Species Definition in Bacteriology[J]. Int.j.syst.bacteriol, 1994, 44(4):846-849.

[6] Wang Q. Naive Bayesian classifier for rapid assignment of rRNA sequences into the new bacterial taxonomy[J]. Appl. Environ. Microbiol, 2007, 73.

[7] Douglas G M, Maffei V J, Zaneveld J R , et al. PICRUSt2 for prediction of metagenome functions[J]. Nature Biotechnology. 2020, 38: 685–688.

[8] Schloss P D, Westcott S L, Ryabin T, et al. Introducing mothur: Open-Source, Platform-Independent, Community-Supported Software for Describing and Comparing Microbial Communities[J]. Applied & Environmental Microbiology. 2009, 75:7537.

[9] Segata N, Izard J, Waldron L , et al. Metagenomic biomarker discovery and explanation[J]. Genome Biology, 2011, 12: R60.

[10] Parks DH, Beiko RG. 2010. Identifying biologically relevant differences between metagenomic communities. Bioinformatics 26:715-721.

[11] Chen MT, Hou PF, Zhou M, et al. Resveratrol attenuates high-fat diet-induced non-alcoholic steatohepatitis by maintaining gut barrier integrity and inhibiting gut inflammation through regulation of the endocannabinoid system. Clinical Nutrition, 2020;39:1264-1275.

[12] Chen MT, Hui SC, Lang HD, et al. SIRT3 Deficiency Promotes High-Fat Diet-Induced Nonalcoholic Fatty Liver Disease in Correlation with Impaired Intestinal Permeability through Gut Microbial Dysbiosis. Molecular Nutrition & Food Research, 2019;63.
