# Supplementary figures and images for: JK5G postbiotics attenuate immune-related adverse events in NSCLC patients by regulating gut microbiota: a randomized controlled trial in China
Source: Front Oncol. 2023 Aug 4;13:1155592. doi: 10.3389/fonc.2023.1155592 (PMC10436471; doi:10.3389/fonc.2023.1155592)

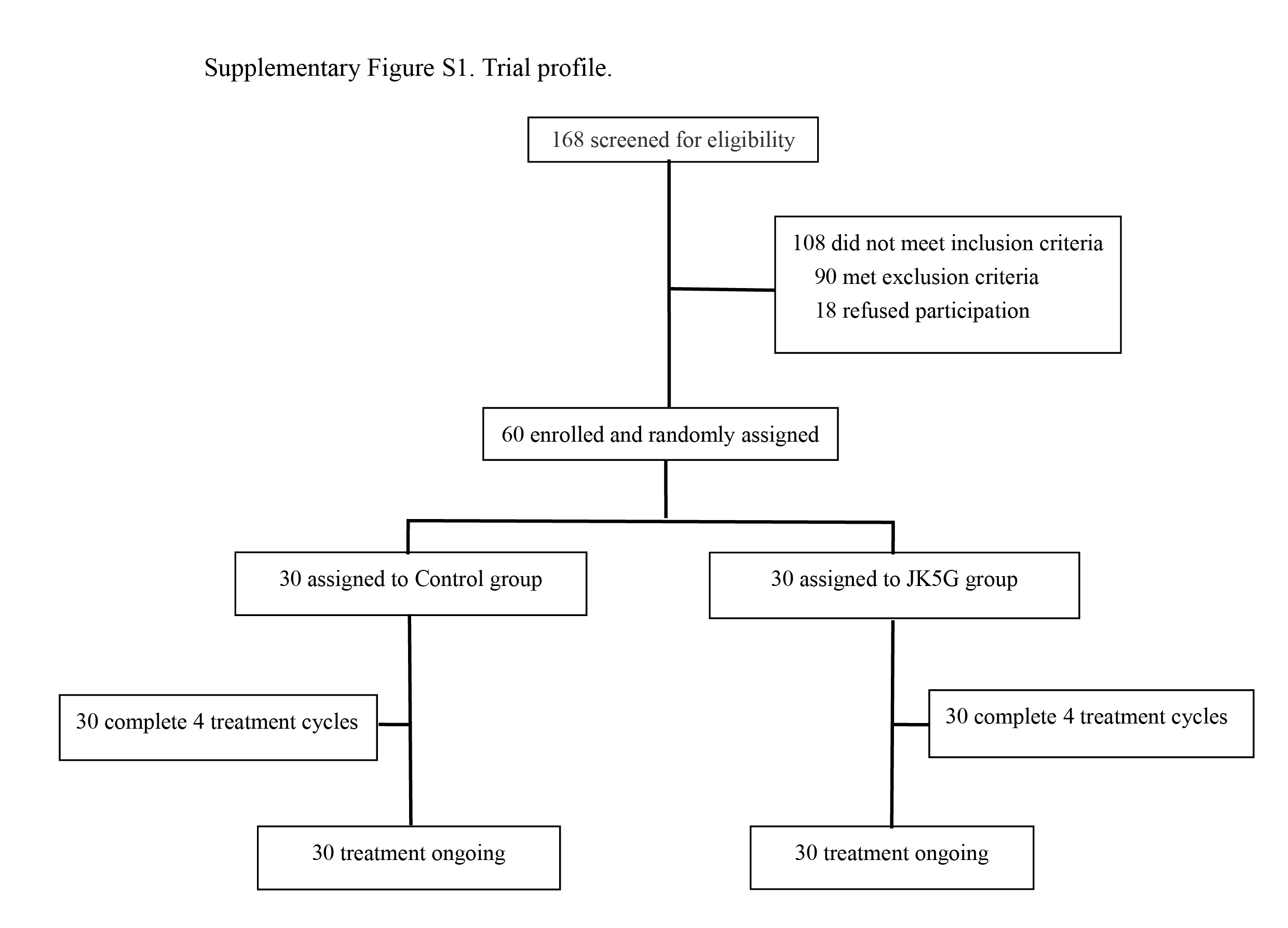

Supplement: Supplementary file 2 [file Image_1.tif]

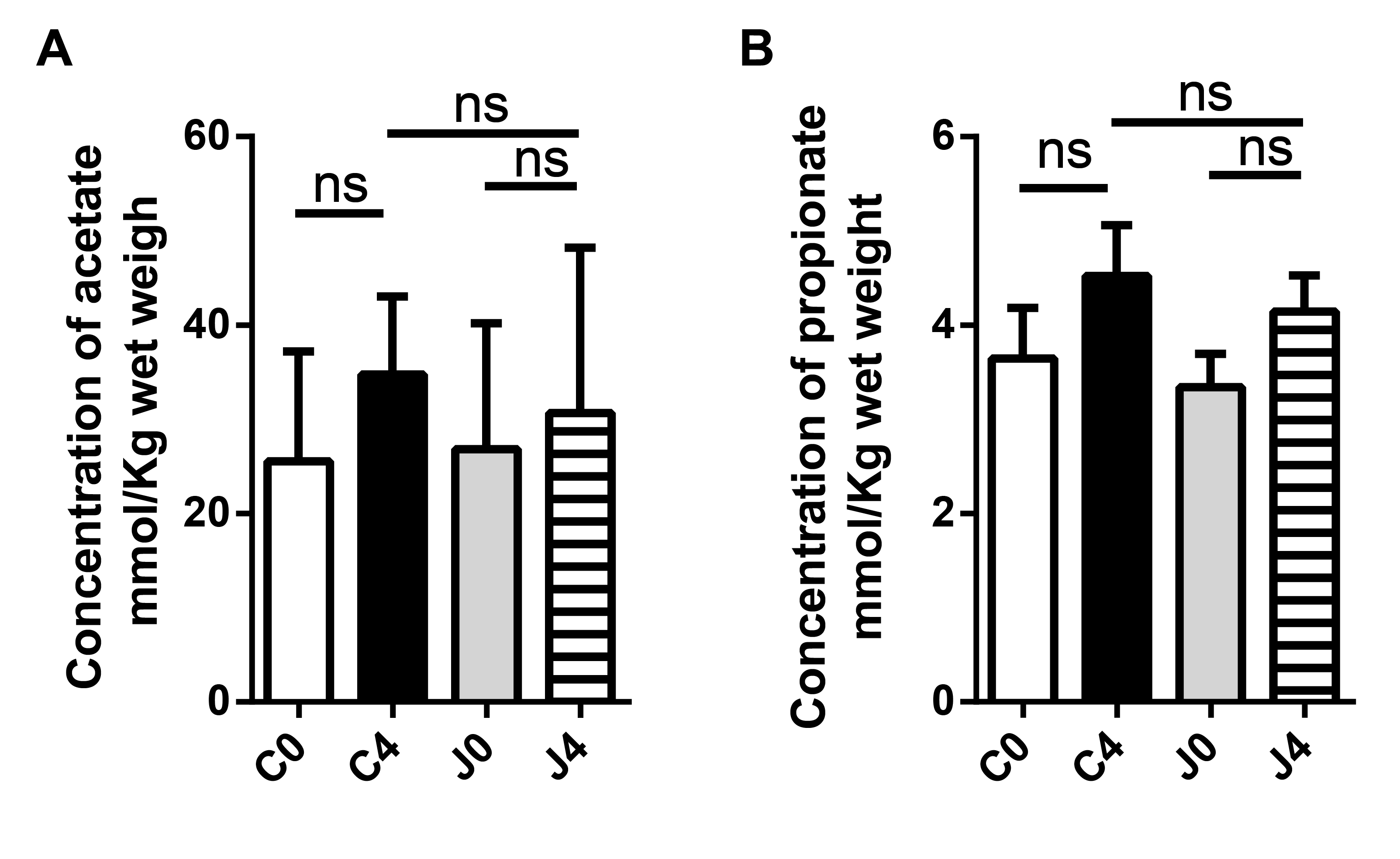

Supplement: Supplementary Figure 2 — Fecal acetate (A) and propionate (B) levels among different groups. Data are reported as means ± SEM; ns, no significance. Multiple groups were tested by one-way ANOVA followed by Bonferroni’s post hoc test. [file Image_2.tif]
